# Health‐related quality of life of type 2 diabetes mellitus patients: A cross‐sectional study in the cape coast metropolis

**DOI:** 10.1002/hsr2.1937

**Published:** 2024-02-25

**Authors:** Leticia Awontayami Amaama, George Nkrumah Osei, Perditer Okyere, Victor Obiri Opoku, Theophilus Junior Yankey, Tetteh Attoh, Mainprice Akuoko Essuman, Jacob Martey, Richard K D Ephraim

**Affiliations:** ^1^ Department of Medical Laboratory Science, School of Allied Health Sciences University of Cape Coast Cape Coast Ghana; ^2^ Department of Internal Medicine, Komfo Anokye Teaching Hospital, College of Health Sciences Kwame Nkrumah University of Science and Technology Kumasi Ghana; ^3^ Department of Physician Assistant Studies, School of Allied Health Sciences University of Cape Coast Cape Coast Ghana

**Keywords:** EQ‐5D‐5 L, Ghana, health‐related quality of life, Type 2 diabetes mellitus

## Abstract

**Background and Aim:**

Type 2 diabetes mellitus (DM) has in recent decades become a global pandemic, accounting for over 90% of DM cases. The study evaluated the health‐related quality of life (HrQoL) and identified its determinants among type 2 DM patients at the University of Cape Coast Hospital.

**Methods:**

We conducted our study at the University of Cape Coast Hospital from January to March 2022. The EQ‐5D‐5L questionnaire was administered to 68 type 2 DM patients. Data were then inputted into Microsoft Excel and analyzed accordingly using IBM SPSS statistical software version 26 and GraphPad Prism 8.

**Results:**

The mean age of the participants was 60.71 ± 12.18 with 55.9% being females. The average systolic, diastolic blood pressure and fasting blood glucose (FBG) of participants were 140.99 ± 22.27, 85 ± 11.14 and 7.97 ± 2.66 respectively. With the EQ‐5D‐5L scale, participants reported severe to extreme problems mainly in pain/discomfort (19.1%) and mobility (8.8%) dimensions. Approximately 21% (14/68) of patients reported themselves as being in perfect health based on the EQ‐5D index score with no significant difference between males and females (*p* ≥ 0.05). On a scale of 0 to 100, most (26.5%) of the participants rated their general health state at 80. Age was significantly associated with all five dimensions while patients with comorbidities had higher odds of experiencing pain/discomfort and anxiety/depression.

**Conclusion:**

The study reveals that pain/discomfort and anxiety/depression are the most experienced problems among patients with type 2 DM. The HrQoL of type 2 DM patients was also found to be affected by age, comorbidities, systolic and diastolic blood pressure. Therefore, identifying these factors and developing appropriate interventions is crucial for improving patient outcomes and enhancing treatment outcomes.

## 1 INTRODUCTION

Diabetes mellitus (DM) is a serious, chronic condition that occurs when the body is unable to produce insufficient amounts of insulin or cannot effectively use the insulin it produces.^1^ Type 2 DM has in recent decades become a global pandemic, accounting for over 90% of DM cases.^2^ Lifestyle modifications, genetic predisposition, aging and obesity are associated with developing type 2 DM.^3^

In contrast to non‐diabetics, both type 1 and 2 DM patients portray a significantly impaired HrQoL which decreases further as disease progresses with complications.^4^, ^5^ Sulphonylureas and insulin used for treating DM pose a threat to hypoglycemic episodes^6^‐ All these complications decrease the quality, duration, and productivity of life in diabetes mellitus patients.^7^ In addition, the non‐stop medical care and lifestyle modifications like a change in diet, and an increase in physical activity cumulatively affect the QoL of the patients (including their physical, and psychosocial well‐being).^8^ The psychosocial and psychological problems associated with DM, as well as the burden of disease and the lifestyle restrictions of DM patients therefore require professional support and specific training to minimize complications and improve their well‐being.^9^, ^10^

Health‐related Quality of life (HrQoL) is a multidimensional and subjective concept that assesses the impact of disease and its treatment across the physical, psychological, and social domains of functioning and well‐being.^9^ Evaluating the HrQoL is essential in assessing the reverberation of a disease from the patient's view providing additional information on laboratory data and subjective symptoms.^10^ Rarely, no studies in Ghana have focused on the HrQoL in diabetic mellitus patients, questioning our holistic approach (supporting patients' physical, mental, social, and spiritual well‐being) to health care. This study therefore evaluated the HrQoL and also identified the factors that determine the HrQoL among type 2 DM patients at the University of Cape Coast Hospital. The findings of this study will excite more strategic policies to be directed at the treatment, management, and delivery of quality care to type 2 DM patients in Ghana.

## 2 METHODOLOGY

### 2.1 Study site, design, duration, and sample size

A cross‐sectional study was conducted at the University of Cape Coast Hospital from January to March 2022. The University of Cape Coast Hospital, located on the University campus, provides services to both the University and the surrounding communities. A non‐randomized sampling approach was used to select and collect data from the consented participants. A total of 68 participants were recruited for the study.

### 2.2 Eligibility criteria

Type 2 DM patients who have been receiving treatment at the diabetes and hypertension clinic of the University of Cape Coast Hospital were eligible for the study. Type 1 DM patients were excluded. Also, type 2 DM patients on any form of dialysis were excluded.

### 2.3 Data collection

#### 2.3.1 Collection of demographic, clinical, and therapeutic data

Demographic data (age, gender, level of education, marital status, and employment), clinical data (duration of diabetes mellitus and presence of comorbidities), and therapeutic data (types of medication and number of medications) were obtained using a structured questionnaire.

#### 2.3.2 Measurement of blood pressure, weight, height, and estimation of body mass index (BMI)

Blood pressure was measured using a mercury sphygmomanometer and a stethoscope. All measurements were done following recommendations of the American Heart Association.^11^ Using a wall‐mounted graduated ruler and weight (to the nearest 0.1 kg) in light clothing, with a weighing balance, the height of participants was measured. The BMI was then calculated as the ratio of the weight (kg) and the square of the height (m^2^).^12^

#### 2.3.3 Blood sample collection and estimation of biochemical parameters

After an overnight fast (8−12 h), 3 mL of venous blood was collected from the participants and analyzed for fasting blood glucose (FBG) and creatinine using a chemistry analyzer (Shenzhen Mindray BS‐120, Shenzhen Mindray Bio‐Medical Electronics Co., Ltd.).

#### 2.3.4 Assessment of quality of life

The EuroQol 5‐dimensional 5‐level (EQ‐5D‐5L) quality assessment scale was used to assess the HrQoL of participants.^10^, ^13^ The EQ‐5D‐5L is a standardized and validated tool used for summarizing HrQoL to estimate Quality Adjusted Life Years (QALYs) and changes in QALYs resulting from health care usage.^14^ The instrument includes a visual analogue scale (EQ‐VAS) which provides a single global rating of self‐perceived health and is scored on a 0−100 mm scale. English version of the scale was used.

### 2.4 Data analysis

Initial entry and organization of data were done using Microsoft Excel. Further analysis and figures were generated using IBM SPSS statistical software version 26 and GraphPad Prism 8. Cronbach *α* was used to check the reliability of the items and domains. Summary statistics were done for the outcome and independent variables and presented as means ± standard deviations (in the case of scale variables) and frequency/percent (in the case of categorical variables). Binary logistic regression analysis was used to identify demographic and clinical factors associated with each domain of HrQoL independently. To do this, all 5 dimensions on the EQ‐5D dimensions were merged and thus dichotomized to “no problem” or “some or extreme problem.” For all analyses, *p* < 0.05 were considered statistically significant.

## 3 RESULTS

### 3.1 Demographics characteristics of study participants

The study recruited a total of 68 participants with a mean age of 60.71 ± 12.18. The majority of participants were married (63.2%), within the ages of 51−70 (61.8%) and females (55.9%) (Table 1). Most of the participants had been diagnosed of having type 2 DM less than a year ago (79.4%) (Table 1). A high occurrence of co‐morbid conditions was also found among study participants (76.5%).

**Table 1:** Sociodemographic characteristics of study participants.

Characteristics	Frequency	Percent
Mean age, *n* (SD)	60.71 ± 12.18	
*Age categories*		
≤50	15	22.1
51−70	42	61.8
>70	11	16.2
*Gender*		
Male	30	44.1
Female	38	55.9
*Employment*		
Unemployed	13	19.1
Informal sector	25	36.8
Formal sector	12	17.6
Retired	18	26.5
*Marital status*		
Single	3	4.4
Married	43	63.2
Widow	19	27.9
Divorced	3	4.4
*Education*		
Basic education	9	13.2
Junior high	15	22.1
Senior high	7	10.3
Tertiary	24	35.3
None	13	19.1
*Duration of diabetes*		
<1year	54	79.4
≥1year	14	20.6
Have comorbidities	52	76.5

### 3.2 Clinical and laboratory characteristics of study participants

The average systolic and diastolic blood pressure of participants were 140.99 ± 22.27 and 85 ± 11.14 respectively (Table 2). The participants had average FBG and creatinine of 7.97 ± 2.66 and 1.17 ± 0.75, respectively (Table 2).

**Table 2:** Clinical and laboratory characteristics of participants.

Variables	Mean ± SD	Participants with results
Systolic Blood Pressure (mg/hg)	140.99 ± 22.27	68 (100)
Diastolic Blood Pressure (mg/hg)	85 ± 11.14	68 (100)
Weight (kg)	73.6 ± 15.9	63 (92.65)
Fasting Blood Glucose (mmol/L)	7.97 ± 2.66	65 (95.59
Height (m)	1.56 ± 0.14	5 (7.35)
Body Mass Index (kg/m^2^)	48.18 ± 5.27	5 (7.35)
Creatinine (mg/dL)	1.17 ± 0.75	29 (42.65)

Abbreviation: SD, standard deviation.

### 3.3 Medication history of participants

The majority of the participants indicated that they have or are taking medications (79.4%). Out of this, 30.9% of the participants were taking two different drugs with the least number of them taking four drugs (4.4%) (Table 3). Most of the participants were taking antidiabetic drugs (77.9%), followed by antihypertensive drugs (13.2%) (Table 3).

**Table 3:** Characteristics of participants based on intake of medication.

Medication history	Number	%
No	14	20.6
Yes	54	79.4
*Number of medicines*		
1	14	20.6
2	21	30.9
3	16	23.5
4	3	4.4
*Drugs taken by respondents*		
Antidiabetics	53	77.9
Antihypertensive	9	13.2
Antidiuretics	3	4.4
NSAIDS	5	7.4
Others^a^	5	7.4

Abbreviation: NSAIDS, Nonsteroidal anti‐inflammatory drugs.

^a^ Others refer to drugs such as statins (2), vitamins (1), benzodiazepines (1) and analgesics (1).

### 3.4 Reliability test of EQ‐5D domain scores of study participant

Four out of five domains had good internal reliability (*α* ≥ 0.7) with Cronbach's *α*: mobility *α* = 0.750, self‐care *α* = 0.753, usual activity *α* = 0.724, pain/discomfort *α* = 0.739 and anxiety/depression *α* = 0.0.807 (Table 4). However, the self‐care domain had the lowest mean score (0.31) with pain/discomfort having the highest mean score (1.35) (Table 4).

**Table 4:** Results of reliability test and EQ‐5D domain scores of study participants.

	Cronbach's *α*	Mean	Standard deviation
Mobility	0.759	0.75	1.028
Self‐care	0.753	0.31	0.758
Usual activities	0.724	0.53	0.954
Pain/discomfort	0.739	1.35	1.219
Anxiety/depression	0.807	1.06	1.006
Overall	0.796	0.8	0.75

### 3.5 Quality of life based on individual EQ‐5D‐5L dimensions

With regard to individual dimension scores on the ED‐5D‐5L scale, participants reported severe to extreme problems mainly in pain/discomfort (19.1%) and mobility (8.8%) dimensions (Table 5). Also, the percentage of participants who reported no problems in the categories of pain/discomfort and anxiety/depression was relatively lower (30.9% and 36.8%, respectively) (Table 5).

**Table 5:** Quality of life based on Individual EQ‐5D dimensions among participants.

	Level of Perceived Problem *n* (%)*				
	1	2	3	4	5
Mobility	40 (58.8)	11 (16.2)	11 (16.2)	6 (8.8)	0 (0.0)
Self‐care	56 (82.4)	6 (8.8)	3 (4.4)	3 (4.4)	0 (0.0)
Usual activities	46 (67.6)	14 (20.6)	4 (5.9)	2 (2.9)	2 (2.9)
Pain/discomfort	21 (30.9)	19 (27.9)	15 (22.1)	9 (13.2)	4 (5.9)
Anxiety/depression	25 (36.8)	20 (29.4)	18 (26.5)	4 (5.9)	1 (1.5)

Abbreviations: 1, No problems; 2, Slight problems; 3, Moderate problems; 4, Severe problems; 5, Extreme problems.

### 3.6 Quality of life based on Europe quality of life—visual analogue scale (EQ‐VAS) score

Table 6 summarizes the proportions of the most reported health states of participants. About 20.6% of participants reported themselves as being in perfect health based on the EQ‐5D index score with no significant difference between males and females (*p* ≥ 0.05). This was followed by participants with slight pain/discomfort and anxiety/depression, with no problems in the other three dimensions (8.8%), and this was higher among female participants (Table 6).

**Table 6:** Quality of Life based on EQ‐VAS score among study participants.

Health states	Gender		Total (*n* = 68)
	Male (*n* = 30)	Female (*n* = 38)	
Without problems in all five dimensions	7 (23.3)	7 (18.4)	14 (20.6)
Slight anxiety/depression without problems in the other four dimensions	2 (6.7)	1 (2.60)	3 (4.4)
Slight pain/discomfort without problems in the other four dimensions	1 (3.3)	1 (2.6)	2 (2.9)
Slight pain/discomfort and anxiety/depression, with no problems in the other three dimensions	1 (3.30)	5 (13.20)	6 (8.8)
Moderate anxiety/depression, slight pain/discomfort without problems in the other three dimensions	3 (10.0)	2 (5.3)	5 (7.4)
Moderate pain/discomfort with no problems in the other four dimensions	2 (6.7)	0 (0.0)	2 (2.9)
Slight problems in all dimensions.	1 (3.3)	1 (2.6)	2 (2.9)
Other states	13 (43.3)	21 (55.3)	34 (50.0)

**Figure 1:**
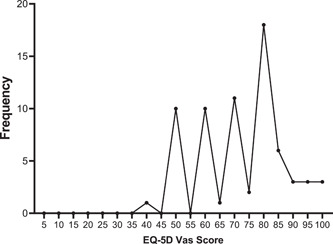
Self‐rated health scores among study participants.

Figure 1 summarizes the self‐reported EQ‐VAS of patients. On a scale of 0−100, a total mean score of 72.5 was observed among participants using the EQ‐5D‐VAS Score. Most (26.5%) of participants rated their general health state at 80. Only one patient rated her health status below 50.

### 3.7 Factors associated with HrQoL based on EQ‐5D dimension scores

The binary logistic regression analysis showed that age was significantly associated with all five dimensions. However, for every increase in age by 1 (year), the odds of a type 2 DM patient experiencing challenges with mobility, self‐care, usual activity, pain/discomfort and anxiety/depression increase by 1.08, 1.08, 1.12, 1.06, and 1.07‐folds, respectively (Table 7). Systolic blood pressure was significantly associated with pain/discomfort and anxiety/depression while diastolic blood pressure was significantly associated with only pain/discomfort (Table 7) Compared to those with comorbidities, those without comorbidities had lower odds of experiencing pain/discomfort and anxiety/depression. However, sex, duration of diabetes, creatinine level, medication intake and FBG were not significantly associated with all five dimensions (Table 7).

**Table 7:** Logistic regression analysis of participants' demographic and clinical characteristics with EQ‐5D dimension scores.

	EQ‐5D dimensions									
	Mobility		Self‐care		Usual activity		Pain/discomfort		Anxiety/depression	
Variables	OR	*p* Value	OR	*p* Value	OR	*p* Value	OR	*p* Value	OR	*p* Value
Age (in years)	1.08 (1.02−1.14)	**0.004**	1.08 (1.00−1.16)	**0.03**	1.12 (1.04−1.19)	**0.001**	1.06 (1.01−1.11)	**0.015**	1.07 (1.02−1.13	**0.004**
Sex (0 = male, 1 = female)	1.40 (0.52−3.73)	0.503	1.13 (0.32−4.0)	0.85	0.92 (0.33−2.56)	0.878	1.62 (0.58−4.57)	0.361	1.28 (0.48−3.45)	0.623
Systolic blood pressure	1.03 (0.99−1.04)	0.082	1.03 (1.00−1.06)	0.08	1.02 (1.00−1.05)	0.057	1.05 (1.02−1.08)	**0.002**	1.03 (1.01−1.06)	**0.014**
Diastolic blood pressure	1.01 (0.97−1.06)	0.534	1.02 (0.97−1.09)	0.42	1.03 (0.98−1.08)	0.265	1.07 (1.01−1.13)	**0.019**	1.01 (0.97−1.06)	0.571
Weight	0.97 (0.94−1.00)	0.082	1.00 (0.96−1.04)	0.97	0.97 (0.94−1.01)	0.15	0.97 (0.94−1.01)	0.119	0.97 (0.93−1.00)	0.063
Fasting blood glucose	0.94 (0.77−1.	0.51	0.90 (0.69−1.18)	0.45	0.97 (0.79−1.12)	0.726	0.91 (0.75−1.10)	0.336	0.90 (0.74−1.08)	0.259
Body mass index	1.12 (0.75−1.65)	0.584	−	−	−	−	1.08 (0.73−1.62)	0.697	−	−
Creatinine	2.0 (0.60−6.45)	0.265	1.08 (0.37−3.14)	0.89	1.77 (0.58−5.45)	0.319	1.36 (0.24−7.84)	0.733	0.67 (0.24−1.90)	0.454
Duration of disease	1.09 (1.00−1.18)	0.044	1.05 (0.97−1.14)	0.23	1.06 (0.98−1.14)	0.157	1.07 (0.97−1.17)	0.182	1.07 (0.98−1.17)	0.146
Have comorbidity (0 = Yes, 1 = No)	0.39 (0.11−1.37)	0.141	0.60 (0.12−3.08)	0.54	0.40 (0.10−1.59)	0.193	0.23 (0.07−0.76)	**0.016**	0.24 (0.08−0.79)	**0.019**
Medication (0 = yes, 1 = no)	3.32 (0.97−11.32)	0.056	1.36 (0.32−5.89)	0.68	2.60 (0.78−8.68)	0.12	3.26 (0.66−16.10)	0.147	0.50 (0.15−1.64)	0.254

## 4 DISCUSSION

DM has become the leading cause of high morbidity and mortality in the 21st century. HrQoL assessment is a significant health outcome representing the ultimate goal of all health interventions. The study evaluated the HrQol and the factors that determine the HrQoL among type 2 DM patients at the University of Cape Coast Hospital.

We found an average age of 60.71 ± 12.18 years among participants, with the majority of participants within the age range of 51−70 years (61.8%). This indicates that the majority of the type 2 DM patients are middle‐aged to older adults. Our finding was slightly lower than that reported in similar studies by Sakamaki et al.,^10^ Ogbonna et al.,^15^ Cardoso et al.,^9^ and Gu et al.^16^ who found an average age range of 63.3( ± 10.3), 65( ± 12.4), 66.28( ± 9.678) and 62.67 years in Japan, Nigeria, Portugal, and China, respectively. Our result was however higher than those observed in similar studies by Nsiah et al.,^17^ Afaya et al.,^18^ Antwi‐Baffour et al.,^19^ and Saleh et al.^20^ who found the mean age to be 51.31 (±0.97), 57.5(±11.8), 45.9 (±14.3) and 54.2 (±11.2) years respectively in Ghana and Bangladesh. These variations could be due to differences in sample size, study population, and duration of data collection.

In agreement with studies by Mata‐Cases et al.^21^ and Parik and Patel^22^ we found a high occurrence of comorbid conditions (76.5%) among study participants. This shows that type 2 DM is not an isolated condition but associated with other health problems. This finding however differed from that of Afaya et al.^18^ who observed a decreased occurrence of comorbid conditions among study participants (39.4%).^18^ This could be due to the study population and sample size employed in the two studies; Our study used a smaller sample size (68) with the majority of participants being older than those in Afaya et al.'s study (sample size = 330). Our findings together with those of Mata‐Cases et al.^21^ and Parik and Patel^22^ in Spain and India respectively stand to indicate that older patients with type 2 DM experience higher occurrences of co‐morbidities than younger patients. Also, having comorbidities was positively associated with pain/discomfort and anxiety/depression in our study. This finding is consistent with studies by Lygidakis et al.^23^ who found comorbidities among type 2 DM patients to have a positive correlation with anxiety/depression thereby decreasing the quality of life among individuals with type 2 DM.

According to our study, extreme problems were found during usual activities (2.9%), pain/discomfort (5.9%), and anxiety/depression (1.5%) among participants. This finding is similar to that of Saleh et al' study which observed extreme problems in usual activities (3.6%), pain/discomfort (15%), anxiety/depression(14.2%), mobility (6.4%), and self‐care (1.2%) among their participants.^20^ This shows that type 2 DM presents challenges beyond blood sugar management, impacting physical well‐being, pain, and mental health even to the extreme level. Also, an increased level of depression/anxiety (63.3%) and pain/discomfort (69.1%) found in our study confirms earlier reports by Saleh et al.,^20^ Parik and Patel,^22^ and Arrieta et al.^24^ This further indicates that anxiety/depression and pain/discomfort are paramount problems among type 2 DM patients.

In comparison with other similar studies by Arrieta et al.^24^ (30.6%) and Cardoso et al.^9^ (33%−87.9%), our study found a lower proportion of participants having no complaint about their health (20.6%). The disparity in the results may be due to the sample size employed in various studies. However, our participants had a higher average self‐rate health score of 72.5 using the EQ‐5D VAS score indicating that participants consider themselves to be in better health with higher overall quality of life. This is similar to the findings of Cardoso et al.,^9^ Sakamaki et al.,^10^ Parik and Patel,^22^ and Arrieta et al.^24^ who found an average EQ‐VAS score of 64.85, 74.3, 78.83, and 79.3 among Portugal, Japan, Indian and Columbia populations respectively. These slight variations in results observed could be attributed to the fact that this measure is subjective and hence relies on individuals' perceptions oftheir health, rather than clinical assessments.

Our study also found age to have an impact on all five dimensions of the EQ‐5D‐5L questionnaire. This slightly differed from the findings of Parik and Patel^22^ who found age to have a negative correlation with a patient's health state and Saleh et al.^20^ who found age to be significantly associated with only self‐care and pain/discomfort.^20^, ^22^ Gender and FBG were not associated with any of the EQ‐5D dimensions among study participants. This reveals that there is no difference in the quality of life between males and females with type 2 DM and that blood sugar levels alone do not predict a patient's HrQoL among type 2 DM.

One of the strengths of our study was the inclusion of reliability testing (Cronbach's *α*) to establish good internal consistency of the EQ‐5D Domain scale among study participants. Additionally, we provided real‐world data regarding the factors affecting type 2 DM patients' HrQoL. This contributed to shedding light on the variables that affect HrQoL in type 2 DM patients to aid in the development of effective interventions to improve HrQoL and related outcomes. Our study had a few limitations. To begin with, our study was a cross‐sectional study and therefore could not evaluate within‐subject changes in quality of life and establish causal relations across different factors. Additionally, our sample size was very small therefore results may not likely be representative of the general population of Ghana.

## 5 CONCLUSION

Most patients with type 2 DM have problems with pain/discomfort and anxiety/depression. Age, comorbidities, diastolic and systolic blood pressure are important factors associated with the Hr‐QoL in patients with type 2 diabetes. Therefore, it is crucial to identify factors that affect the HrQoL of type 2 DM patients to help develop appropriate interventions for both clinicians and health policymakers to implement and improve the HrQoL in these patients.

## AUTHOR CONTRIBUTIONS

**Leticia Awontayami Amaama**: Conceptualization; data curation; methodology; visualization; writing—original draft; writing—review and editing. **George Nkrumah Osei**: Conceptualization; methodology; visualization; writing—original draft; writing—review and editing. **Perditer Okyere**: Conceptualization; methodology; writing—review and editing. **Victor Obiri Opoku**: Data curation; writing—review and editing. **Theophilus Junior Yankey**: Data curation; writing—review and editing. **Tetteh Attoh**: Data curation; writing—review and editing. **Mainprice Akuoko Essuman**: Formal analysis; visualization; writing—review and editing. **Jacob Martey**: Data curation; writing—review and editing. **Richard K D Ephraim**: Conceptualization; methodology; writing—original draft; writing—review and editing.

## CONFLICT OF INTEREST STATEMENT

The authors declare no conflict of interest.

## ETHICS STATEMENT

Ethical approval was sought from the Institutional Review Board of the Ghana Institute of Management and Public Administration. Informed written consent was also obtained from the participants before recruitment, after giving a detailed education on the basis, aims and justification of this study. All methods were carried out following relevant guidelines and regulations. Confidentiality was also observed throughout the entire study.

## TRANSPARENCY STATEMENT

The lead author George Nkrumah Osei affirms that this manuscript is an honest, accurate, and transparent account of the study being reported; that no important aspects of the study have been omitted; and that any discrepancies from the study as planned (and, if relevant, registered) have been explained.

## Data Availability

Corresponding author had full access to all of the data in this study and takes complete responsibility for the integrity of the data and the accuracy of data analysis.
